# Tramadol-induced addiction shows sex-dependent variation response in pubescent adolescent Wistar rats

**DOI:** 10.1016/j.ibneur.2025.10.007

**Published:** 2025-10-20

**Authors:** Stève Brunel Kenfack Ngoufack, Gwladys Temkou Ngoupaye, Jospin Chirac Noubouwo, Aurelien Fossueh Foutsop, Tatiana Bibiane Diebo Kom

**Affiliations:** a Department of Animal Biology, Animal Physiology and Phytopharmacology Research Unit, University of Dschang, P.O. Box 67, Dschang, Cameroon; b Department of Biological sciences, Faculty of Science, University of Maroua, P.O. Box 814, Maroua, Cameroon

**Keywords:** Tramadol, Pubescent adolescent, CPP, Dopamine, Oxidative stress

## Abstract

Tramadol is an opioid commonly prescribed for the treatment of moderate to severe pain and its abuse creates addiction. According to WHO, 36 million people worldwide suffer from disorders related to drug abuse. Among the consume substances, opioid such as Tramadol, is one of the most used. Therefore, the aim of this study was to evaluate gender variation in response to tramadol abuse. Tramadol (50 mg/kg, p.o) was administered to 28 Wistar rats from both sexes (14 males and 14 females) to induce addictive behavior and was assessed for 12 days on the conditioned place preference (CPP) test. Hyperactivity and risk taking linked to addiction were also assessed on the Elevated plus maze (EPM). On day 12 one hour after the CPP, animals were sacrificed by cervical dislocation and, striatum and hippocampus were collected for biochemicals assay (dopamine, GABA, nitric oxide and malondialdehyde). Tramadol intake induced dependency to tramadol and addictive seeking behavior in CPP in both sexes. Hyperactivity and risk taking assessed on EPM were present in both sexes. Tramadol increased dopamine and reduced GABA in both sexes and increased the malondialdehyde and nitric oxide production. Generally, variations were greater in females. So, these results suggest that pubescent female adolescents showed greater susceptibility to Tramadol abuse induced addictive behavior than males at least in the basic parameters assessed.

## 1 Introduction

Tramadol is known as a synthetic opioid agent used for the treatment and prevention of mild to severe pains (Matthiesen et al., 1998). It is broadly used as analgesic agent which acts as an µ-opioid receptors agonist (Jasim et al., 2022). The long-term use of Tramadol has been associated with addiction and increase nitric oxide, oxidative stress and apoptosis (Dal et al., 2006, Khatmi et al., 2022). Studies have reported that women have low threshold for pain than men and are at greater risk for developing different forms of chronic pain (Fillingim, 2002, Gaumond and Marchand, 2009). Furthermore, studies have shown that females are less sensitive to analgesic opioids such as Tramadol, Morphine, Methadone, and Heroin when compared to males (Craft, 2008, Dai et al., 2008, Lee and Ho, 2013). Similar findings were observed in rodent models, where male rats exhibit greater analgesia response than female rats to equal doses of opioids (Kepler et al., 1991, Cicero et al., 1997; Satyam et al., 2018). Interestingly sex differences are not limited to pain perception but may also extend to the response to analgesics abuse such as addiction. As such Morphine, Methadone, Heroin which has previously shown less analgesia in female showed a marked sensitivity on females compared to males to develop drug addiction (Carroll et al., 2002, Wang et al., 2006, Brazile et al., 2024). A remarkable observation is that, although the prevalence of addiction is lower in women than men, it was interesting to notice that the difficulty of treatment is more important on women due to the number of relapses observed (Fonseca et al., 2021). Even supposing, the analgesic Tramadol effects have been shown as well as its sensitivity related to sex-gender in pain related disorders (Satyam et al., 2018), understanding its sensitivity regarding sex-gender response in tramadol induce addiction will surely benefit substance abuse management on sex-gender base (Becker and Chartoff, 2019). In Cameroon, the national anti-drugs committee reported that 21 % of the population had used elicit substances, and amongst that 60 % are youths (Metuge et al., 2021). Furthermore, studies conducted in the city of Buea (South West region, Cameroon) estimated the rate of drug abuse among young students at 29.9 % (Metuge et al., 2021). According to this study, tramadol commonly called “Tramol” (with a rate of 2.8 %) was one of the three most abused substances just ahead Cannabis (with a rate of 2.0 %). Finally, there is growing evidence suggesting that adolescent humans and rodents experience many similar structural and functional changes in the brain as they progress to adulthood. For example, dopamine D1 and D2 receptor levels reach a peak and then decline over adolescence (Teicher et al., 1995, Andersen and Teicher, 2000), suggesting that brain development during adolescence is likely similar in many ways between humans and rodents (Schramm-Sapyta et al., 2009). The role of dopamine transmission is key in mediating reward-seeking actions which underpins addiction. Thus, this preliminary study sought to assess males and females’ susceptibilities to tramadol induce addictive behavior based on basic parameters related to addiction, dopamine, GABA, NO and MDA in pubescent adolescent rat model.

## 2 Materials and methods

### 2.1 Drugs and chemicals

Tramadol Hydrochloride was manufactured from Denk Pharma in Germany and administered at the dose 50 mg/kg.

### 2.2 Animal material and ethical statement

Male and female Wistar rats aged 40–42 days and 30–32 days respectively were used at the beginning of the test. The age chosen correspond to the periods of entry into the respective pubescent phase of each sex (Schneider, 2013). The animals were bred and raised at the Animal Physiology and Phytopharmacology Research Unit of the University of Dschang. Animals were housed in a controlled environment at 24 ± 1^°^C with 12:12 h light/dark cycle (lights on at 7:00 AM and off at 7:00 PM). Three animals were housed per cage with free access to water and food. Prior to the experiments, animals were acclimatized to the room for 7 days. To minimize stress, animals were handled gently by the same experimenter throughout the study. During dosing procedures, animals were habituated to the handling for 4 consecutive days beforehand, and efforts have been made to minimize animal suffering as much as possible using atraumatic techniques with minimal restraint. The animals were treated in accordance with the guidelines of the bioethics committee and NIH care.

### 2.3 Animals treatment

A total of 28 rats, 14 males and 14 females, were used. The animals were divided into four groups (administration volume 10 ml/kg) over a period of 12 days following the conditioned place preference (CPP) test model:

• Two vehicle groups (received distilled water by oral route): male vehicle (VEH(M)) and female vehicle (VEH(F))
• Two experimental groups (received Tramadol at the dose 50 mg/kg by oral route): male Tramadol (Trama (M)) and female Tramadol (Trama (F))

#### 2.3.1 Conditioned place preference test

The CPP had two rooms (dark room and lighted room) of equal dimensions, 30 × 30 × 60 cm (length, width, height). The walls and floors of the rooms were different (white wall and smooth floor covered with a plastic mat for the lighted room; black wall and rough floor for the dark room). The two rooms communicated through a door measuring 10 cm × 14 cm (width, height) (Jiang et al., 2016, Alhassen et al., 2021, Barbosa et al., 2023).

The CPP took place in 3 successive stages:

• Pre-conditioning phase or acclimation phase (3 days): during which the doors were opened, the animal had free access to both compartments for 15 min and the initial preference was calculated as: preference index = **Tc / Tc +Ts** (**Tc**: time spent in the light compartment; **Ts**: Time spent in the dark compartment)
• The preconditioning preference index was calculated here as the average of the daily preference indices; To avoid bias, animals with a natural preference for the lighted room were removed from the test.
• Conditioning phase (8 days) where the doors were closed: distilled water was administered in the morning (between 8 a.m. and 9 a.m.) and animals were individually and immediately placed in the black compartment. Six hours later in the afternoon (between 2 p.m. and 3 p.m.), Tramadol was administered and the animal placed in the lighted room. The time spent in each compartment was 30 min. On day 2 the animals received Tramadol in the morning and distilled water 6 h later in the afternoon and the procedure was alternated like this until day 8. In the vehicle group, Tramadol was replaced by distilled water (distilled water in the morning and distilled water in the afternoon).
• Test or post-conditioning phase (1 day): with the doors opened, the animal had the freedom to move between the different compartments for 15 min. As at the end of the preconditioning, the preference index was determined.

The CPP score showing the change in preference was calculated as follows:

#### 2.3.2 CPP score = postconditioning preference index - preconditioning preference index

On day 12, 1 h after the CPP postconditioning test, elevated plus maze (EPM) was performed. One hour after the EPM, the animals were sacrificed by cervical dislocation; the striatum and hippocampus, were taken for the evaluation of dopamine, GABA, MDA and NO levels.

#### 2.3.3 Elevated plus maze

The Elevated plus maze (EPM) was performed to assess hyperactivity and risk-taking in animals. It is a device with two open arms and two closed arms. The test lasted 5 min during which the animal had the opportunity to explore the paradigm. The parameters taken were the time spent and the number of entries into the open and closed arms. Additionally, the number of rearing in the closed arms coupled to the number of head dipping in the open arms were also recorded (Iñiguez et al., 2009).

### 2.4 Biochemical analysis

The striatum and hippocampus were isolated for biochemical analysis. Right striatum and right hippocampus served for dopamine assay; and left Striatum and left hippocampus to other biochemical assays (GABA, MDA and NO).

#### 2.4.1 Homogenates preparation for dopamine assay

The right striatum and right hippocampus were ground (5 % w/v) with grinding solution (37 % HCl solution + Butanol). The ground material obtained was centrifuged at 2000 rpm for 10 min. After centrifugation, 500 µl of the supernatant was introduced into a tube containing 1250 µl of heptane and 155 µl of 0.1 M HCl. The mixture was stirred for 10 min and centrifuged again at 2000 rpm for 10 min. After centrifugation, two phases were obtained. The organic phase from above was taken and discarded and the aqueous phase (200 µl) was stored in Eppendorfs for the dopamine assay (Schlumfjf et al., 1974).

#### 2.4.2 Homogenates preparation GABA, MDA and NO assays

Left striatum and left hippocampus were individually ground to homogenate (10 % w/v) using 0.1 M phosphate buffer containing 1 % Triton-100X (pH 7.4). These homogenates were centrifuged for 15 min (3000 rpm) at room temperature, and 700 µl of the supernatants were collected for various assays (Ngoupaye et al., 2017).

#### 2.4.3 Determination of dopamine level

Dopamine is oxidized in the presence of iodine, for chromophores which absorb at the wavelength 375 nm. During the process, 200 µl homogenate, 50 µl of 0.4 M HCl and 100 μl of sodium acetate buffer were successively introduced into the reaction medium. Subsequently, 100 µl of 0.1 M iodine was introduced and the timer was started for 120 s (2 min). At the end of 2 min, the reaction was stopped by introducing 100 μl of sodium sulfite into the medium. Then the clock was starting again for 90 s. After 90 s, 100 μl of 10 M acetic acid was added to the medium. The tube was then brought to a water bath (100°C for 6 min), then cooled for 2 min in running water. The dopamine concentration was expressed for each sample in ng /mg of protein (Schlumfjf et al., 1974, Kom et al., 2025).

#### 2.4.4 Determination of GABA level

GABA was evaluated according to the method described by Moto et al., (2018) and was based on the coloration formed by the reaction of GABA and ninhydrin in an alkaline medium in the presence of glutamate. In the reaction medium consisting of 200 µl of 0.14 M ninhydrin prepared in carbonate bicarbonate buffer (0.5 M, pH=9.9) and 100 µl of glacial trichloroacetic acid (TCA) at 10 %, 100 µl of homogenate was introduced, and the entire solution was incubated at 60 °C for 30 min. After cooling, each tube’s content was introduced into tubes containing 5000 µl copper tartrate. The tubes were then incubated at 25 °C for 10 min. The absorbance was then read on a spectrophotometer at 451 nm against the blank and was proportional to the concentration of GABA in the sample. The concentration of GABA in each sample was expressed in µg/mg of tissue (Moto et al., 2018).

#### 2.4.5 Determination of malondialdehyde level

A volume of 250 µL of the supernatant was introduced into test tubes. 250 µl of 20 % trichloroacetic acid (TCA), 500 µl of 0.67 % thiobarbituric acid (TBA), and 10 µl of 0.1 % BHT (Butylated hydroxytoluene) were added to each tube. The blank solution consisted of all the above-stated elements except the homogenate. The tubes were sealed and incubated for 10 min at 90 °C and then cooled with tap water. They were then centrifuged at 3000 rpm for 10 min at room temperature. The supernatant was pipetted, and the absorbance read at 532 nm on a BIORAD spectrophotometer, SMART SPEC 3000 (USA), against the blank, and results were expressed in nmol/mg of wet tissue (Wilbur et al., 1949).

#### 2.4.6 Determination of NO level

In the different tubes, 200 μl of tissue homogenate followed by 200 μl of 1 % sulfanilamide (prepared in 5 % orthophosphoric acid) were introduced. In the white tube the homogenate was replaced by the grinding solution (PBS + Triton-X). After mixing, the whole was left away from light for 5 min. Then 200 µl of naphthylethylene diamine (NED1 % prepared in tris- hydroxyl methylamine) were added to the reaction medium and the whole left once again protected from light for 5 min. Once the 5 min are up, the Optical density were read at the wavelength 546 nm. The NO rate was determined from the equation of the calibration curve previously established from the different concentrations of Na2NO. The Griess reaction takes place in an acidic environment and in the absence of light. The concentration of NO in each sample was expressed in µmol/mg (Sreejayan and Rao, 1997).

## 3 Statistical analysis

The results obtained were analyzed using Graph Pad Prism software (version 8.01). They were presented as mean ± Standard Error on Mean (SEM). The Normal distribution of data was assessed using the Shapiro-Wilk normality test. When the data showed normal distribution, parametric tests were used.Values were compared using *t*-test, one-way and two-way analysis of variance (ANOVA) tests, and where differences existed, the Newman Keuls and Bonferoni multiple comparison tests were used to separate means. Values were considered statistically significant at p < 0.05. In order to highlight the sex effect, the global variation of each parameter was calculated as follows:

Δx = (female Tramadol treated-group mean - vehicle female mean) - (male Tramadol treated-group mean - vehicle male mean)

## 4 Results

### 4.1 Effect assessed in the CPP and EPM

#### 4.1.1 Effect on preference in the CPP

**Fig. 1:**
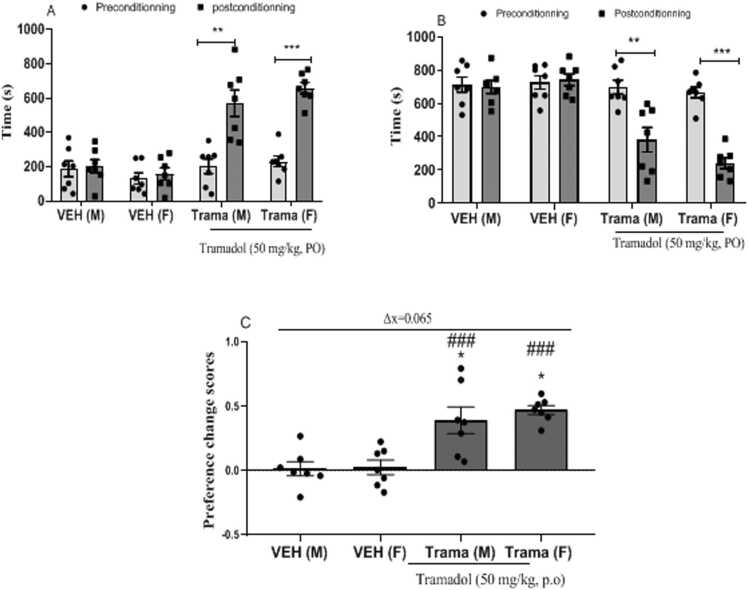
Response variation related to sex on tramadol - abuse induced addiction in the CPP test. A= time spent in the lighted room, B= time spent in the dark room, C= CPP score; Data expressed as mean ± standard error of the mean; n = 7; ** p < 0.01, ***p < 0.001 when compared preconditionning to postconditionning (a and b), Two way Anova followed by Bonferonni test; * p < 0.05, compared to the male vehicle, ### p < 0.001 compared to the female vehicle, paired *t*-test. VEH (M): Vehicle male; VEH (F): female vehicle; Trama (M): Tramadol 50 mg/kg on male; Trama(F): Tramadol 50 mg/kg on female.

Fig. 1 presents effect on Tramadol evaluated on both sexes in Conditioned place preference test (CPP)

Fig. 1A illustrates the time spent in the lighted room (paired with Tramadol) during the preconditioning phase and during the postconditioning phase. The male (VEH (M)) and female (VEH (F)) vehicles showed no variation in the time spent in the lighted room between preconditioning and postconditioning. There was a significant increase in time spent in the lighted room during postconditioning compared to preconditioning in male (Trama M) [F _(1,24) =_ 12.86; p = 0.0015] and female (Trama F) [F _(1,24) =_47.36; p < 0.0001] from Tramadol treated-groups.

Fig. 1B shows the time spent in the dark room (paired with distilled water) during the preconditioning phase and during the postconditioning phase. Male (VEH (M)) and female (VEH (F)) vehicles showed no variation in time spent in the dark room between preconditioning and postconditioning. There was a significant decrease in time spent in the dark room during postconditioning compared to preconditioning in male (Trama M) [F _(1,24) =_10.12; p = 0.0040] and female (Trama F) [F _(1,24) =_ 63.37; p < 0.0001] from Tramadol treated-groups.

The CPP preference change scores in animals are illustrated in the Fig. 1C. No significant difference was observed between male and female vehicles. At same, male Tramadol treated-group presented no significant difference with female Tramadol treated-group. Male Tramadol treated-group (Trama (M)) showed a significant increase on CPP score compared to VEH (M)[t_(6)=_3.361;p = 0.0152] and VEH (F)[t_(6)_= 3.501, p = 0.0128].The CPP score on female Tramadol treated-group (Trama (F)) showed a significant increase compared to VEH (M) [t(_6)=_12.17; p < 0.0001] and to VEH(F) [t_(6)=_13.14;p < 0.0001]. Finally, the average difference in CPP score between Tramadol treated-group and vehicle was 0447 in females and 0382 in males i.e. a global variation of 0065 in favor of females.

#### 4.1.2 Effect on hyperactivity and risk-taking in the EPM

**Fig. 2:**
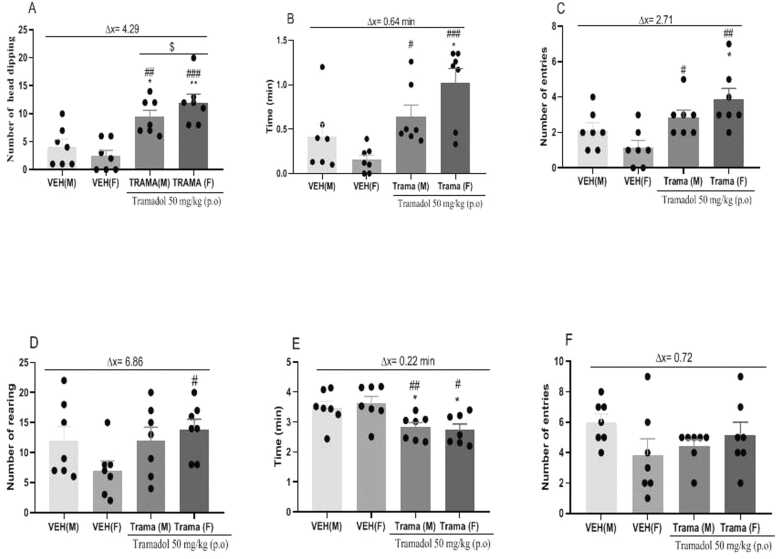
Response variation related to sex on tramadol - abuse induced addiction in the EPM test. A= number of head dipping, B= time spent in the opens arms, C= number of entries in the opens arms, D= number of rearing, E = time spent in the closed arms, F= number of entries in the closed arms; Data expressed as mean ± standard error of the mean; n = 7; * p < 0.05, **p < 0.01, compared to male vehicle; # p < 0.05, ## p < 0.01, ### p < 0.001 compared to the female vehicle; $: comparison between the VEH(M) and VEH(F) paired *t*-test. VEH (M): Vehicle male; VEH (F): female vehicle; Trama (M): male negative control (Tramadol 50 mg/kg); Trama(F): female negative control (Tramadol 50 mg/kg).

The effects of Tramadol in the EPM test are illustrated in the Fig. 2.

The Fig. 2A illustrates the number of head dipping realized by the animals. No significant difference was noted between the male vehicle (VEH(M)) and the female vehicle (VEH(F)). However, a significant increase [t_(6)_= 2.580, p = 0.0418] in the number of head dipping performed by animals in the female Tramadol treated-group (Trama(F)) was observed compared to the male Tramadol treated- group (Trama(M)). The number of head dipping was significantly elevated in the female Tramadol treated-group compared to the female [t_(6)_= 4.819, p = 0.0029] and male [t_(6)_= 3.862, p = 0.0083] vehicles. Similarly, in the male Tramadol treated- group, the number of head dippings performed was significantly elevated compared to the male [t_(6)_= 2.601, p = 0.0406] and female [t_(6)_= 3.381, p = 0.0148] vehicles. The mean difference in the number of head dipping between Tramadol treated-group and the vehicle in females was 9.57 while it was 5.28 in males; giving an overall variation of 4.29 in favor of females.

Fig. 2B presents the time spent by animals in the open arms of the EPM. The male vehicle (VEH(M)) and the male Tramadol treated- group (Trama(M)) showed no significant difference compared to the female vehicle (VEH(F)) and the female Tramadol treated-group (Trama(F)), respectively. An increase in the time spent in the open arms was observed in the female Tramadol treated-group compared to the male [t_(6)_= 3.448, p = 0.0137] and female [t_(6)_= 5.823, p = 0.0011] vehicles. Male animals receiving Tramadol had a non-significant increase in the time spent in the open arms compared to the vehicle. However, a significant increase [t_(6)_= 2.838, p = 0.0297] in the time spent in the open arms was noted in the male Tramadol treated- group compared to the female vehicle. After calculating the mean difference in time spent in the open arms, the values obtained were 0.22 min in males and 0.86 min in females. The overall variation in time spent in the open arms was therefore in favor of females with a value of 0.64 min.

The number of entries into the open arms (Fig. 2C) of the EPM did not significantly changed in the male vehicle and the male Tramadol treated- group compared to the female vehicle and the female Tramadol treated-group respectively. The female Tramadol treated-group showed a significant increase [t_(6)_= 4.478, p = 0.0042] in the number of entries into the open arms compared to the female vehicle. Similarly, compared to the female vehicle, the male Tramadol treated-group showed a significant increase [t_(6)_= 3.032, p = 0.0230] in the number of entries into the open arms. However, the male Tramadol treated-group showed non-significant [t_(6)_= 0.9564, p = 0.3758] increase in the number of entries into the open arms compared to the male vehicle. The mean difference in the number of entries into the open arms between the Tramadol treated-group and the vehicle was 0.71 in males and 2.71 in females. Entries into the open arms were in favor of females, for an overall difference of 2 entries.

Fig. 2D shows the results of the number of rearings performed by the animals. No significant difference was noted between the male vehicle and the female vehicle. Similarly, the number of rearings did not vary between the male Tramadol treated-group and the female Tramadol treated-group. The animals in the male Tramadol treated-group showed no significant difference compared to the male and female vehicles. In females treated with Tramadol, the number of rearings significantly [t_(6)_= 3.667, p = 0.0105] increased compared to females vehicle, but the increase was not significant [t_(6)_= 2.083, p = 0.0824] when compared to males vehicle. The average difference in the number of rearing between the Tramadol treated-group and the vehicle was 0.00 in males and 6.86 in females, for an overall variation of 6.86 rearing in favor of females.

The time spent by animals in closed arms is presented in Fig. 2E. Animals in the female vehicle and female Tramadol treated-group showed no significant difference compared to the male vehicle and male Tramadol treated-group control respectively. In females, animals receiving Tramadol showed a significant decrease in time spent in closed arms compared to the female vehicle [t_(6)_= 2.941, p = 0.0123] and the male vehicle [t_(6)_= 3.497, p = 0.0129]. Similarly, the male Tramadol treated-group showed a significantly lower time in closed arms compared to the male vehicle [t_(6)_= 3.026, p = 0.0232] and the female vehicle [t_(6)_= 3.894, p = 0.0080]. The mean difference in time spent in closed arms between the Tramadol treated-group and vehicle was 0.65 in males and 0.87 in females. The overall variation between sexes was in favor of females with a value of 0.22 min.

Fig. 2F shows the number of entries into the closed arms of the EPM. No significant difference was noted in the vehicle and the Tramadol treated-group in males compared to females. In males only, a reduction, although not significant [t_(6)_= 2.185, p = 0.0715], in the number of entries into the closed arms was observed in Tramadol treated-group compared to the vehicle. In females, no significant difference was noted between the female Tramadol treated-group and the female vehicle. Between the Tramadol treated-group and the vehicle, the mean difference in time in the closed arms was 1.57 in males and 0.72 in females. We therefore note a variation in favor of males of 0.85.

### 4.2 Effect on dopamine and GABA concentrations in the hippocampus and striatum

**Fig. 3:**
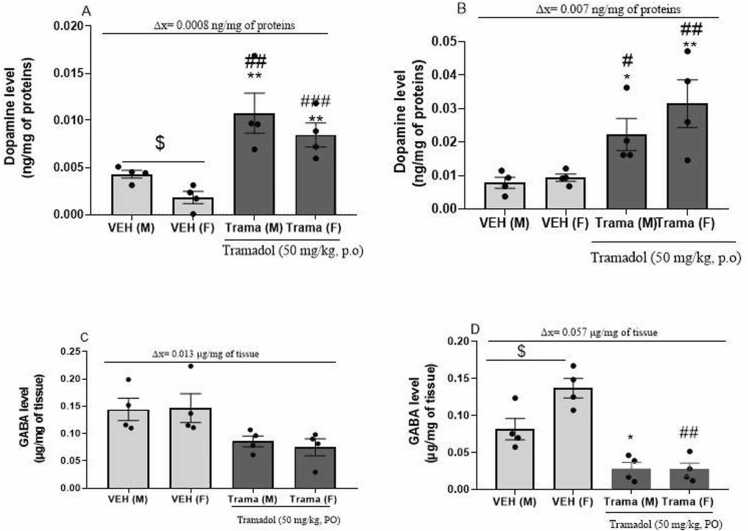
Variation in dopamine and GABA levels induced by Tramadol - abuse in both sexes. A= concentration of dopamine in the hippocampus, B= concentration of dopamine in the striatum, C = GABA concentration in the hippocampus, D= GABA concentration in the striatum; n = 4. Data expressed as mean ± standard error of the mean; * p < 0.05, **p < 0.01, compared to male vehicle; # p < 0.05, ## p < 0.01 compared to the female vehicle; $: comparison between the VEH(M) and VEH(F) paired *t*-test. VEH(M): Vehicle male; VEH (F): female vehicle; Trama (M): Tramadol 50 mg/kg on male; Trama(F): Tramadol 50 mg/kg on female.

Fig. 3 highlights the effect of Tramadol on dopamine and GABA levels in the hippocampus and the striatum.

The concentration of dopamine in the hippocampus was illustrated in the Fig. 3A. There was a significant decreased [t _(3) =_2.626; p = 0.0786] at the baseline level of dopamine in female (VEH (F)) compare to male (VEH (M)) vehicles. The male Tramadol treated-group did not present a significant difference in comparison to female Tramadol treated-group. The dopamine concentration significantly increased in the male Tramadol treated-group (Trama (M)) compared to VEH (M) [F_(2,9)_ = 9.343;p = 0.0064] and VEH (F) [F_(2, 9)_ = 14,88; p = 0,0014] and in the female Tramadol treated-group (Trama (F)) in comparison to VEH (F) [F_(2,9)_= 17,98; p = 0,0007] and VEH(M) [F_(2,9)_ = 15.38;p = 0.0012]. The overall variation of dopamine increase induced by tramadol between Tramadol treated-group and vehicle was 0.0066 ng/mg of proteins for females and 0.0058 ng/mg of proteins for males. The global variation of dopamine in the hippocampus was 0.0008 ng/mg of proteins in favor of females.

Fig. 3B shows the concentration of dopamine in the striatum. Dopamine concentration significantly increased in Trama (M) [F _(2, 9)_ = 4.298; p = 0.0490] and Trama (F) [F _(2, 9)_ = 9.263; p = 0.0065] compared to VEH (M) and VEH (F) respectively. In the striatum, no significant difference between male and female vehicles; as well as between the male Tramadol treated-group and the female Tramadol treated-group was noted. However, the average difference of striatal dopamine between Tramadol treated-group and vehicle in female was 0.022 ng/mg of proteins and 0.015 ng/mg of proteins in males. The calculation of global variation was 0.007 ng/mg of proteins in favor of females.

There was an overall tramadol effect in the GABA level (Figs. 3C and 3D) within groups in the hippocampus [F _(3,12)_ = 3.969; p = 0.0354] 2 and the striatum [F _(3,12)_ = 8.062; p < 0.0001].

Fig. 3C shows the variation in the amount of GABA in the hippocampus. Tramadol induced a tendency to a significant decrease in GABA concentration in both the male (Trama (M)) [t _(4.375)_ = 2.573; p = 0.0567] and the female (Trama (F)) [t _(4.846)_ = 2.355; p = 0.0668] compared to their respective vehicles. These changes lead to an average reduction of 0.059 μg/mg of tissue in males and 0.072 μg/mg of tissue in females i.e a hippocampal GABA global variation of 0.013 μg/mg of tissue.

The variation in GABA level in the striatum are illustrated in Fig. 3D. There was a significant increase in GABA concentrations in female vehicle (VEH (F)) compared to male (VEH (M)) [t _(3)_ = 4.568; p = 0.0197]. But no significant difference was observed between TRAMA(M) and TRAMA(F). Tramadol induced a significant decrease in the quantity of GABA in Trama (M) [t_(3)_ = 4.226; p = 0.0242] and Trama (F) [t_(3)_ = 7.222; p = 0.0055] in comparison with VEH (M) and VEH (F) respectively. The average difference of GABA decrease induced by tramadol in male versus female in the striatum was 0.053 μg/mg of tissue in males and 0.110 μg/mg of tissue in females. Thus, the global variation between males and females was 0.057 μg/mg of tissue in favor of females.

### 4.3 Effect on MDA and Nitric oxide levels in the hippocampus and striatum

**Fig. 4:**
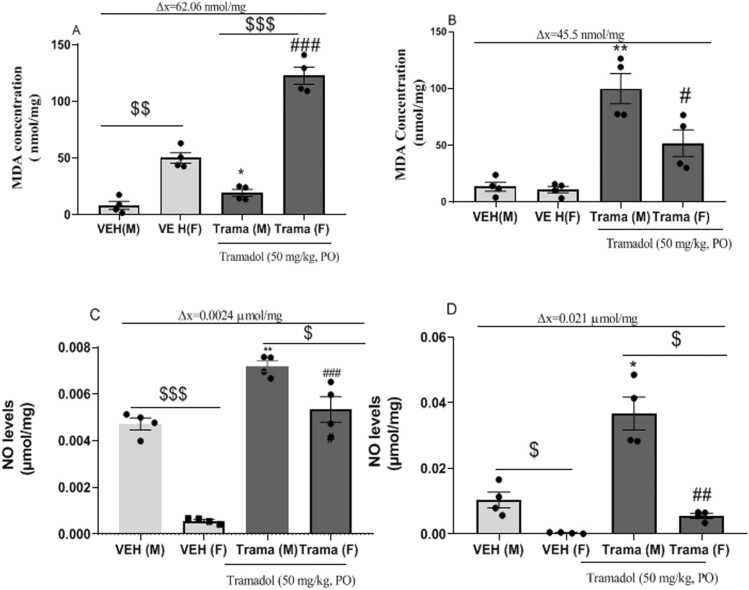
Variation in MDA and NO concentrations induced by Tramadol - abuse in both sexes. A= MDA concentration in the hippocampus, B = MDA concentration in the striatum, C = NO concentration in the hippocampus, D = NO concentration in the striatum; n = 4 Data expressed as mean ± standard error of the mean; * p < 0.05, **p < 0.01, ***p < 0.001, compared to the male vehicle; #p < 0.05, ## p < 0.01, ### p < 0.001 compared to the female vehicle. $ p < 0.05, $$ p < 0.01, $$$ p < 0.001 comparison between the VEH(M) and VEH(F) or TRAMA(M) and TRAMA (F), paired *t*-test. VEH(M): Vehicle male; VEH (F): female vehicle; Trama (M): Tramadol 50 mg/kg on male; Trama(F): Tramadol 50 mg/kg on female.

Figs. 4A and 4B show the variation in the concentration of malondialdehyde (MDA). There was an overall tramadol effect in the hippocampus [F _(3,12)_ = 105.2; p < 0.0001] and the striatum [F _(3,12)_ = 20.76; p < 0.0001]. Fig. 4A shows malondialdehyde concentrations in the hippocampus. There was a significant increase in MDA concentration baseline in the female vehicle (VEH (F)) compared to male vehicle (VEH (M)) [t_(3)_ = 6.882;p = 0.0063]. At same, the quantity of MDA was significant increased on female Tramadol treated-group in comparison to the male Tramadol treated-group (t_(3)_= 14,07; p = 0,0008). The quantity of MDA showed a tendency to a significant increase [t_(5.920)_ = 2.377;p = 0.0556] in the male Tramadol treated-group (Trama(M)) compared to the male vehicle (VEH(M)). Female exposed to tramadol (Trama (F)) showed a significant increase in MDA compared to their vehicle (VEH (F)) [t_(3)_ = 12.84;p = 0.0010]. The average differences of MDA concentration were 11.04 nmol/mg in males and 73.1 nmol/mg in females ie a variation of 62.06 nmol/mg in favor of female.

Fig. 4B shows MDA concentrations in the striatum. No significant difference was observed between male and female vehicles. At same, male Tramadol treated-group presented no significant difference with female Tramadol treated-group. MDA concentration significantly increased in Trama (M) [t_(3)_ = 7.533.84;p = 0.0048] and Trama (F) [t_(3)_ = 3.410;p = 0.00143] in comparison to VEH (M) and VEH (F) respectively. In males, the average difference was 86.4 nmol/mg while in females it was 40.9 nmol/mg. Finally, the MDA concentration variation was in favor of males i.e 45.5 nmol/mg.

Figs. 4C and 4D shows the effect of sex on tramadol response at the level of NO concentration. There was a tramadol effect within the groups in the hippocampus [F _(3,12)_ = 104.5; p < 0.0001] and the striatum [F _(3,12)_ = 33.68; p < 0.0001].

Fig. 4C shows the concentration of NO in the hippocampus. NO concentration baseline in male vehicle (VEH (M)) was significantly increased compared to female vehicle (VEH (F)) [t_(3)_ = 13.92;p = 0.0008]. At same, male Tramadol treated-group was presented a significantly increase of NO concentration in comparison to the female Tramadol treated-group [t_(3)_ = 3.264; p = 0.0047]. There was a tramadol effect in both sexes as there was a significant increase in male Tramadol treated-group (Trama (M)) [t_(3)_ = 6.067;p = 0.0090] and female Tramadol treated-group (Trama (F)) [t_(3)_= 8.315, p = 0.0036] compared to their respective controls. There increase of NO levels was 0.0024 μmol/mg in males and 0.0048 μmol/mg in females. The final variation of NO levels was 0.0024 μmol/mg in favor of females.

Fig. 4D shows NO concentration in the striatum. There was a significant decrease in female vehicle (VEH (F)) and female Tramadol treated-group compared to male vehicle (VEH(M)) and male Tramadol treated-group respectively [t_(3)_ = 4.388;p = 0.0219; t_(3)_= 5.696;p = 0.0107]. Regardless of sex there was a significant increase in NO concentration in males [t_(3)_ = 3.973;p = 0.0285] and in females [t_(3)_ = 6.609; p = 0.0071] compared respectively to male and female vehicles. The average differences were 0.026 nmol/mg in males and 0.005 nmol/mg in females i.e a global variation of 0.021 nmol/mg in favor of females.

## 5 Discussion

Although, studies have reported Tramadol abuse in adult rodent males (Cha et al., 2014), there is not yet studies reporting sex response to Tramadol abuse in males and females. Indeed, there is growing recognition of sex differences in the behavior and biology of opioid exposure and use that are important for addressing more effective treatment approaches (Lee and Ho, 2013, Becker and Chartoff, 2019). Gutierrez and Taffe (2024) in their studies on heroin tolerance showed that adolescent females are more affected by opioid than adults, highlighting the fact that adolescence is a critical neurodevelopmental period that is associated with heightened risk-taking behavior, including experimentation with intoxicating substances (Miech et al., 2023, Temourian et al., 2023). This justifies our studies that sought to assess males and females’ susceptibilities to tramadol induce addictive behavior based on basic parameters related to addiction, dopamine, GABA, NO and MDA in pubescent adolescent rat model.

The difference in male and female susceptibility to Tramadol addiction was determined using the CPP behavioral test which is the largely use to evaluated the reward properties of drug (Blanco-Gandía et al., 2018). In sexes, between the preconditioning (before drug administration) and postconditioning phases (after drug administration), Tramadol increased the time spent in the lighted room (associated with Tramadol) compared to the time spent in the dark room (associated with distilled water). Based on different time obtained, the initial preference of the animals was thus reversed with a high preference change score. These results are in agreement with Cha et al. (2014) studies on adult mice who demonstrated that Tramadol reversed preference. Furthermore, the comparative study between the sexes showed a marked change in preference change score in females than in males. These results are in agreement with studies of Hamor et al. (2023) and Ryan et al. (2018) respectively carried out on Morphine and Oxycodone, two opioids receptors agonists which demonstrated that CPP change preference score are greater in female than male. The underlined greater changes in females compared to males were related to estradiol that enhances the motivation for drugs abuse by interacting with reward system as previously shown by Silverman and Koenig, 2007 in their studies on amphetamine addiction. Additionally, addiction is also related to hyperactivity and higher risk taking as mentioned in the studies of Faron-Górecka et al., (2004) Tramadol induces an increase in hyperactivity. In this study, Tramadol successfully induced hyperactivity on animals which was mainly manifested by an increase in the time taken, the number of entries and the number of head dippings in the open arms of the EPM. These results are in agreement with the studies of Bush and Vaccarino (2007), Famitafreshi and Karimian (2020) and Ngoufack et al., (2025) which showed that significant animal activity in the open arms of the EPM was observed in subjects addicted to drugs such as cocaine, morphine and nicotine. Similarly, with the CPP test, greater hyperactivity was observed in females compared to males, this further highlighting the probable role of estrogens in the development of addiction in females. Indeed, studies by Bo et al., (2021) successfully demonstrated that estradiol was responsible for an increase in the time spent and the number of head dippings in the open arms of the EPM and that this reflected hyperactivity and increased risk-taking in females. After assessing the behavioral preferences and the hyperactivity of subjects through the conditioned place preference test and the EPM test, particularly noting that females exhibited a stronger preference and hyperactivity compared to males, it is essential to explore biochemical assays to track the biochemical changes underlining the behavioural modifications.

In both pain and addiction, studies have reported that Tramadol impacts dopaminergic and gabaergic circuits, as well as on the synthesis of nitric oxide and lipid peroxidation (Abdel-Zaher et al., 2011, Panadanabiona, 2017, Li et al., 2020). Consequently, sex sensitivity associated to Tramadol on striatal and hippocampal concentrations of dopamine, GABA, MDA and NO was also evaluated. In both sexes, Tramadol increased striatal and hippocampal dopamine concentration. These results are in agreement with Velásquez et al. (2019) who showed dopamine increase after opioid administration on rat. Dopamine concentration (striatum and hippocampus) in response to Tramadol effect was more marked in females than males confirming the behavioural results previously observed. Indeed, several studies have reported that estrogen is able to enhance de production of proteins such as DAT (dopamine protein transporter), tyrosine hydroxylase (dopamine synthesis enzyme) and upregulate D1 receptors (Chavez et al., 2010, Johnson et al., 2010), enhancing the effect of opioid on increasing dopamine production. Addiction process also requires inhibition of gabaergic signalling normally responsible for blocking dopamine exaggerated production in the reward circuit (Lemière, 2016). Tramadol reduced the amount of GABA in the hippocampus and striatum in response to the respective increase in hippocampal and striatal dopamine concentration. This reduction is more important on females than males agreeing with the results obtained by Velásquez et al. (2019) which observed a marked decrease in GABA concentration due to Morphine addiction in female compared to male adults, suggesting that estrogen increases the sensitivity of opioid receptors may contribute to reduce GABA production induced by Tramadol abuse in females. Similarly, Huang and Woolley (2012) and Tabatadze et al. (2015) in their studies on estrogen effect proved that in female neurons, ERα activates mGluR1a which enhances excitatory signal and contribute to decreasing presynaptic GABA neurotransmission. These justify our data showing that addictive effect of opioids is marked on females. While the sex effect has been observed in specific neurotransmitter (dopamine, GABA), it is also crucial to examine how this variable may influence oxidative stress levels, paving the way for a more comprehensive understanding of biological interactions.

Previous studies have also reported that Tramadol and others drugs abuse such as nicotine significantly increased MDA and NO concentrations (Mohamed and Mahmoud, 2019, Xia et al., 2020, Adeluyi et al., 2019). In this study Tramadol effectively increased MDA and NO production. The literature review carried out by de Toda et al. (2023) re-sensed a set of works which proved that in the normal condition males display a higher production of ROS and less efficient antioxidant (GSH, Catalase and superoxide dismutase) in brain which contribute to enhance MDA and NO production. Interestingly, after Tramadol administration the present results show higher striatal and hippocampal NO concentrations in females than in males, and so was the MDA concentration in the hippocampus. Previous studies mentioned that estrogens influence the NO system in both peripheral and nervous tissues (Farsetti et al., 2009). Indeed, estrogen induced nNOS ARNm increase which induces the production of high amount nNOS enzyme responsible to NO production on Morphine analgesia process (Hosseini et al., 2011). Thus, estrogen could have potentiated the effect of Tramadol in the production of NO.

Higher NO level is capable to react to superoxide anion and produce peroxynitrite ion (ONOO-). The peroxynitrite ion is responsible to the increase of lipid peroxidation and its final product, the Malondialdehyde (MDA) (Chen et al.,2012). Excess Dopamine levels is also able to increase lipid peroxidation and MDA concentration (Shah et al., 2012). Studies done by LaVoie and Hastings (1999) on Methamphetamine abuse had proved that elevated levels of dopamine can undergo autoxidation in striatum, leading to the formation of toxic quinones. The cycling of these quinones contributes to oxidative stress, primarily through the production of superoxide radicals and hydrogen peroxide leading to increased lipid peroxidation and MDA production. Likewise, MDA higher production on female is due to an elevated concentration of dopamine in female hippocampus that triggers the process of lipid peroxidation. These findings justify the higher concentration of NO and hippocampus MDA in female than male. However, unlike the hippocampus, the concentration of MDA in the striatum of male rats was higher than in female rats. Our data showed that baseline MDA striatal concentration in male and female vehicles were approximatively similar. However, at equal concentrations, since antioxidants have been shown to be lower in males, we can assume that males were not able to cope with the exaggerated increase in MDA due to the consumption of Tramadol. This would explain that despite the effects of estrogens which predispose to the deleterious effects of addiction, the concentration of MDA in striatum still remains higher in males than females. In general, these results firstly show that Tramadol induces addiction. It is worth remembering that the concentration of estrogens has been shown to be at its peak phase during the pubescent period which may have contributed to aggravating the onset of addiction in pubescent females. The same observation concerning the sensibility of females is also observed on adult females which have been shown to be more sensitive to opioid. Our results depict that Tramadol shows a greater sensitivity in adolescent females compared to males in the biochemicals parameters assessed.

## 6 Conclusion

The present data demonstrated in the parameters studied that Tramadol - abuse induced addiction in both male and female adolescent pubescent animals, with a marked sensitivity to females. Tramadol abuse increased dopamine, reduced GABA, and increased MDA and NO production. We noticed a marked sensitivity to Tramadol abuse in females compared to males in all the behavioural and biochemicals parameters assessed. These preliminary results therefore align with others studies on opioids in females with consideration of sex and ovarian hormones suggesting a consequent management in term of treatment based on sex-gender variation on Tramadol abuse. However, more studies are needed to further establish this sex-dependent variation response.

## Author contributions

SBK Ngoufack co-designed the work, and conducted the laboratory trials, data analysis, and manuscript writing as part of its PhD thesis. GT Ngoupaye designed the work and supervised the experiments, data analysis, and manuscript writing. Materials preparation and data collection were performed by SBK Ngoufack assisted by JC Noubouwo, TD KOM, AF Foutsop, and supervised by GT Ngoupaye. All authors read and approved the final manuscript.

## CRediT authorship contribution statement

**Bibiane Tatiana Diebo Kom:** Software, Methodology. **Aurelien Fossueh Foutsop:** Writing – review & editing, Software. **Gwladys Temkou Ngoupaye:** Writing – review & editing, Supervision, Software, Resources, Project administration, Methodology, Data curation, Conceptualization. **Stève Brunel Kenfack Ngoufack:** Writing – original draft, Methodology, Investigation, Conceptualization. **Jospin Chirac Noubouwo:** Software, Resources, Investigation.

## Ethical statement

Animal experiments were carried out in accordance with the guidelines of the Cameroon Bioethics Committee (No. FWA-IRB00001954) and the international principles of laboratory animal protection (NIH Publication 8023, revised 1996).

## Compliance with ethical standards

Animal experiments were carried out in accordance with the international principles of laboratory animal protection (NIH Publication 8023, revised 1996), and use of laboratory animals’ manual, and Directives 2010/63/EU for animal experiments. Efforts have been made to minimize animal suffering as much as possible.

## Conflicts of Interest

The authors have non-financial interests to disclose.
